# Hybrid Wavelet–ML models for regional drought forecasting in Norway

**DOI:** 10.1038/s41598-025-22416-1

**Published:** 2025-11-04

**Authors:** Türker Tuğrul, Sertaç Oruç, Jessica Louise Hall, Ali Ulvi Galip Şenocak, Mehmet Ali Hınıs

**Affiliations:** 1 Technology Faculty, Civil Engineering Department, Gazi University, Ankara, Türkiye; 2 The Center for Sámi Studies, UiT Norges Arktiske Universitet, Tromsø, N-9037 Norway; 3 Faculty of Engineering and Natural Sciences, Department of Civil Engineering, Ankara Yıldırım Beyazıt University, Ankara, Türkiye; 4 The Arctic Youth Network and The Foundation for Law and International Affairs, Washington, USA; 5 Faculty of Engineering, Civil Engineering, Aksaray University, Aksaray, Türkiye

**Keywords:** LSTM, XGBoost, SVM, Catboost, Wavelet transformation, MLP, Climate sciences, Environmental sciences, Hydrology, Natural hazards

## Abstract

Drought is a natural disaster that often remains unnoticed until ecosystem impacts become severe. Therefore, monitoring and detecting droughts are important research topics. Consequently, drought indices with different focuses, such as precipitation or soil moisture, have been developed. Yet, the utility of the indices is limited before the beginning of the drought. To overcome this shortcoming, drought forecasting and providing decision-makers with an early warning to mitigate the effects is an important research topic. This study aims to take on the forecasting of the droughts with its novelty on the spatial focus, Norway (Drammen, Hamar, and Lillehammer). We forecast the Effective Drought Index (EDI) across spatially diverse Norwegian regions without hydrological constraints. To achieve this, we have utilized precipitation data between 1980 and 2025 and trained our machine learning models, namely, Support Vector Machine (SVM), Multi-layer Perceptron (MLP), Extreme Gradient Boosting (XGboost), Long-Short Term Memory network (LSTM), and Categorical Boosting Algorithm (Catboost). Moreover, the latent feature space is extended by wavelet transformation (WT). The innovative aspect of this study and its contribution to the literature is its novel application of the WT to some algorithms. Furthermore, unlike the literature, EDI was chosen as the drought index in this study, further increasing its innovative nature. Our results indicate that long short-term memory networks enhanced by wavelet transformation provide the best forecasts. Here, the best performance, LSTMW-M04, is achieved over Drammen (*r* = 0.9765, NSE = 0.9510, KGE = 0.8641, PI = 0.3211, and RMSE = 0.2207). Although LSTM is already an innovative and successful algorithm, we have further improved the model performance. This result will help decision-makers in a future drought study with both the model input structure and the algorithm used.

## Introduction

 Droughts are considered as one of the most severe and complex forms of natural hazards; these climate related events cause catastrophic consequences which have an impact on ecosystems, food security and agriculture worldwide^1^. Droughts are a variable type of natural hazard that take shape in many forms and are commonly identified by their intensity, frequency and length^2^. Meteorological drought, one form of drought, is routinely defined by a significant reduction in precipitation over a specific amount of time^3^. When such conditions endure, the resulting lack of moisture gradually reduces soil water availability, which in turn disrupts crop growth and agricultural productivity. This progression illustrates how sustained meteorological drought can trigger agricultural drought, establishing a clear chain of impact between atmospheric conditions and land-based consequences^4^. Another form of drought is “hydrological drought”^5^ which is the result of long-term imbalances in water flow and access to water, caused by periods of below average precipitation. These often last more than nine months and can severely impact the behavior of rivers, lakes and groundwater systems^6^. A reduction in precipitation can lead to environmental disturbances that can impair socioeconomic activities^7^.

Due to the continuous intensification of climate change, the frequency, length and severity of droughts are predicted to continually increase, leading to heightened risks to ecosystems as well as to humanity^8,9^. The threat posed by droughts is a matter of global concern; although some regions are affected more severely than others, the increase of drought intensity worldwide highlights the urgent need for universally coordinated responses^7,10^. Undoubtedly, there is a need to develop multitiered water management strategies^7^ as many communities are vulnerable due to increasing challenges to their food security, livelihoods and economic stability^11,12^.

Norway is often perceived as a country with an abundant water supply due to its rivers, lakes and glacial reserves which contributes both to the country’s natural beauty and to its development of energy infrastructure. Although this is the case, over the last few years, there has been an increase in the frequency of dry spells and summer droughts in southeastern and central regions of Norway^13^. This pattern is part of a heightened probability for larger water deficits in southeastern Norway whereby local water resources are put under pressure by prolonged periods of low precipitation and a consequent reduction in soil moisture^14^. This trend has created notable challenges for agriculture, ecosystems and water management in the region. Consequently, a nation long regarded as having a stable and abundant supply of water is now increasingly confronting periods of drought. There is a need for localized drought monitoring and forecasting that ensures consistent and reliable data collection, identifying drought patterns and formulating coherent data sets to be utilised.

There are many different drought indices in existence, that have been developed to allow for the monitoring of droughts. The Standardized Precipitation Index (SPI) and the Palmer Drought Severity Index (PDSI) are both used to help quantify the severity, length and spatial extent of moisture deficits. The SPI largely focuses on precipitation changes over fluctuating time scales whilst the PDSI uses both precipitation and temperature data to evaluate the cumulative impact on soil moisture. This makes the PDSI a useful index to understanding agricultural and hydrological drought^14,15^. However, the Effective Drought Index (EDI) is unique in that it offers an advantage by capturing both the duration and intensity of precipitation deficits^16^, which ensures its usefulness in the context of short - to medium-term drought detection. Developed in the late 1990s by Byun and Wilhite^16^, the EDI was a response to the perceived limitations of current drought indices. The EDI goes beyond the SPI and PDSI as it uses daily precipitation data as well as incorporating the cumulative precipitation deviation from the mean over time. EDI can be calculated both daily and monthly. Its greatest advantage over other drought indices is its greater resolution in identifying droughts, allowing it to produce more meaningful results than other indices. One of its greatest advantages is its ability to consider one-year calculations, particularly in its daily calculations. Many researchers in the literature have cited its reliability and ability to identify significant droughts. In addition, the need for a single dataset in its calculations increases the interest of researchers in EDI^17^.

Machine learning (ML) and deep learning (DL) have become useful tools in hydrometeorological forecasting because of the increase in the computational power and the data availability. It is argued that these new approaches are outperforming traditional, statistical methods because of their ability to model complex, nonlinear and high-dimensional relationships between climactic variables^18^. ML and DL models can capture subtle patterns and temporal dependencies that may have been overlooked by conventional models, which improves predictive accuracy and reliability^19^.

Ensemble learning techniques are well-suited for environmental datasets. These include eXtreme Gradient Boosting (XGBoost), and Categorical Boosting (CatBoost). They are well suited to environmental datasets as they can handle structured tabular data as well as managing missing values^20–22^. Building on this, Support Vector Machines (SVMs) are efficient when used in high-dimensional spaces. These have been used successfully in classification and regression tasks related to drought monitoring^23^. Networks such as Multi-Layer Perceptrons (MLP) and Long Short-Term Memory (LSTM) are effective tools for capturing temporal dependencies in sequential data. This is essential for time series-based drought prediction^24^.

By combining these models together, a strong foundation is created that can in turn be used to develop data-centered and regionally adaptive drought forecasting systems. This is particularly evident when combined with drought indices such as the Effective Drought Index (EDI) as this joint approach accounts for both short- and long-term precipitation deficits^16^. The integration of drought indices has the potential to inform early warning systems as well as water resource management in the face of increasing climate instability.

In recent years there has been an increase in the use of machine learning (ML) and deep learning (DL) techniques in drought prediction. Scholars argue that these newer methods have demonstrated substantial improvements in terms of accuracy and responsiveness, when compared to traditional statistical approaches^18^. Many studies have utilized ML/DL models with standard drought indices, such as SPI, but there is a noticeable lack of research that centers on the EDI combined with ML/DL methods. This is particularly evident in the context of Norway.

Many researchers are using machine learning methods to predict future EDI scenarios. EDI is attracting attention because it offers more effective solutions than SPI. Piri et al.^25^ developed drought prediction models for the Iran region using various drought indices, including EDI. In their study, they used SVM as the machine learning method and utilized optimization techniques. Another study on the future prediction of EDI is by Deo et al.^26^. In their study, the Artificial Neural Networks (ANN), SVM and the Extreme Learning Machine (ELM) were used as machine learning methods and WT was used as the data preprocessing method. Another study is of Deo and Şahin^27^. They used EDI with machine methods, Extreme Learning Machine (ELM) and Artificial Neural Networks (ANN), without pre-processing data. When examining studies used not only for EDI but also for SPI estimation, models were generally developed using two or three machine learning techniques. Furthermore, a single scenario was generally applied to the model inputs^23,28,29^. In this study, however, four different model input structures were used. Considering the literature, it was observed that the number of previous studies using LSTM, MLP, Xgboost, Catboost and SVM algorithms simultaneously are limited. Additionally, the innovative aspect of this study is enhanced using WT. This study aims to fill this gap in the literature.

Norway’s diverse climatic zones, differing topography and hydrological characteristics necessitate regionally specific drought forecasting models. However, the majority of the existing literature on ML- and DL-based drought prediction has focused on regions that are prone to severe water scarcity, such as North America, South Asia and parts of the Mediterranean^23^. There has been little exploration of this in a Norwegian context. This is a notable point when developing models that are specifically tailored to individual climates that include the agricultural identity of different regions of the country. In many data-based modeling studies in the literature, region of interest selected as areas suffering from drought^30–32^. This study examines regions of Norway that have so far remained largely unaffected by severe droughts and, as a result, have been neglected in most research. Yet, these areas are not immune and could experience both positive and negative impacts from drought. For example, some agricultural lands covered by glaciers may be restored to agriculture due to drought. Based on all these factors, this study selected specific regions in Norway that further enhances the innovative nature of this article.

This study aims to fill this research gap. This shall be done by introducing the Effective Drought Index (EDI) into a Norwegian context. As noted previously, the EDI has been used in many other geographical locations as an appropriate indicator of both short- and long-term drought conditions. However, its application in a Norwegian context is limited, the integration of EDI with ML and DL techniques is still widely underexplored. This is particularly so in the context of regionally specific forecasting which is needed in Norway. This research utilizes and compares a suite of advanced ML and DL algorithms: XGBoost, CatBoost, Support Vector Machines (SVM), Multi-Layer Perceptrons (MLP), and Long Short-Term Memory (LSTM) networks, evaluates their relative performance and explores how these methods can be calibrated to Norway’s diverse climatic and agricultural zones.

This study’s unique contribution is found in its regional specificity by applying predictive models across three climatically distinct cities - Lillehammer, Hamar and Drammen. By doing this, the study adds to the scientific field by advancing the scientific understanding of drought dynamics in high-latitude regions as well as advancing the practical capacity for localized, anticipatory water management systems. The insights from this study will be particularly of interest and value for sectors such as agriculture, energy and municipal planning as these requireupdated and accurate drought forecasts to inform adaptive decision-making. Thus, this study will fill a methodological and geographic gap in the literature, laying the groundwork for futureresearch on Nordic drought resilience. This research also offers an applicable and usable framework for similar analyses in other vulnerable, climate-sensitive regions.

## Methodology

This work presents an analysis of EDI data derived from three distinct locations of Norway. The analysis used a range of machine and deep learning methods implemented within different model architectures. In this study, the algorithms implemented include Long-Short Term Memory network (LSTM), Support Vector Machine (SVM), Extreme Gradient Boosting (XGBoost), Cat Boosting (CatBoost), Multilayer Perceptrons (MLPs).

Upon completing the analyses, wavelet transformation (WT) was paired to enhance the performance of the results.

### Study region and data

**Fig. 1:**
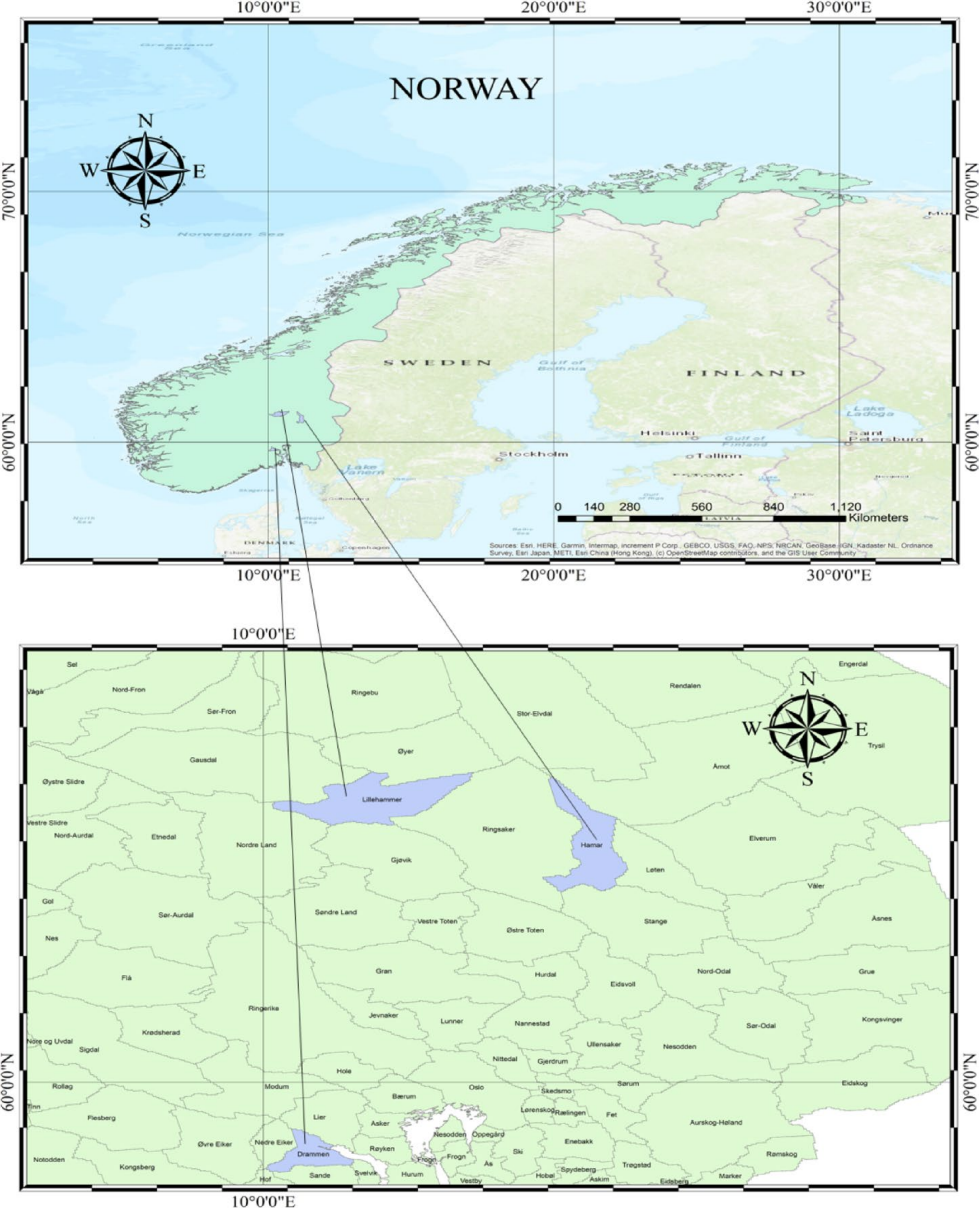
Study area. The maps were generated using ArcMap (version 10.8) from ArcGIS Desktop (https://desktop.arcgis.com).

The study considers three diverse regions across Norway, each characterized by distinct geographical and climatic conditions that influence their drought forecasting needs (Fig. 1). These regions include Lillehammer, Hamar, and Drammen were selected to contrast inland continental and coastal settings. Lillehammer and Hamar lie in the interior (Gudbrandsdal and Hedmark) where relatively dry continental climate supporting grain and dairy production with Hamar, surrounding areas being among Norway’s most fertile agricultural lands. Drammen situated southwest of Oslo along the Drammensfjord located in southeastern coastal regions, experience milder climate and nearby fertile lowlands, supporting horticulture, vegetable farming, and grain cultivation; however, while not a particular concern for Drammen, though past land-use policies have led to concerns about soil erosion from intensified arable farming as with many urbanizing areas^33–37^. Monthly precipitation was used to calculate EDI data of these three cities are given in Table 1 together with the coordinates of the stations. Across these regions, agriculture remains largely small-scale and part-time and shifting climatic patterns with urban development shaping the future of agricultural production systems and land use.

Missing data were minimal (0.6%) and filled by linear interpolation. Data normalization was carried out by min-max procedure (Eq. 1).

1 $$\:X^{\prime\:}=\frac{X-{X}_{min}}{{X}_{max}-{X}_{min}}$$

**Table 1:** Data statistics.

Data point	Start	End	Temporal Resolution	Min. (mm)	Max. (mm)	Latitude	Longitude
Drammen	1980-1	2025-3	Monthly	5.6	458.5	59.7432	10.1933
Hamar	1980-1	2025-3	Monthly	7.0	348.1	60.7940	11.0664
Lillehammer	1980-1	2025-3	Monthly	8.0	375.9	61.1141	10.4613

### Effective drought index (EDI)

The Effective Drought Index (EDI) is first introduced by Byun and Wilhite^16^. This index quantifies drought (or wetness) on a daily (or monthly) basis by comparing current effective precipitation to its climatological mean and standard deviation. Compared to other drought indices that rely on long accumulation windows (e.g., SPI), EDI responds quickly to emerging dry or wet spells while still incorporating prior moisture conditions.

Effective Precipitation (EP).

For a target month (day) t, each month’s (day’)s precipitation in the antecedent window is weighted inversely by its temporal distance (i.e., more recent rainfall has greater influence).

If $\:P_{t-i}$ is the precipitation that occurred i months before t:

2 $$\:E{P}_{t}={\sum\:}_{\left\{i=1\right\}}^{\left\{N\right\}}\frac{P_{t-i}}{i}$$

where N is the chosen memory length (commonly 365 days for daily EDI or 12 months for monthly EDI). This weighting scheme mimics soil-moisture recession, giving emphasis to recent rainfall.

Climatological Mean and Standard Deviation of EP.

Compute long-term statistics for the same calendar day (or month) over the full record:

3 $$\:{EP}_{m}=\frac{1}{Y}{\sum\:}_{\left\{y=1\right\}}^{\left\{Y\right\}}{EP}_{m,y\:}$$

4 $$\:{\sigma\:}_{EP,m}=\sqrt{\frac{1}{Y-1}{\sum\:}_{\left\{y=1\right\}}^{\left\{Y\right\}}{\:}_{{\left({{EP}_{m,y\:}-\:EP}_{m\:}\right)}^{2}}}$$

where EP_m, y_ is the effective precipitation for calendar month m in year y and Y is the number of years.

EDI Standardization

5 $$\:{EDI}_{t}=\frac{{{EP}_{t\:}-\:EP}_{m\:}}{{\sigma\:}_{EP,m}}$$

### Long-Short term memory network (LSTM)

The Long Short-Term Memory (LSTM) model is an improvement over the standard Recurrent Neural Network (RNN) that effectively can address the vanishing gradients problem. Proposed by Hochreiter and Schmidhuber^37^, LSTM incorporates mechanisms that allow it to process information across extended time periods. The strength of the LSTM lies in its ability to maintain long-term data sequences through improved memory components, such as memory cells and various gates, making it more efficient for handling extended sequences compared to standard RNNs^38^.

The architecture of an LSTM network includes a sequence input (SI) layer which is essential for feeding time series data into the network. This layer connects to the LSTM layer, composed of units that feature an input gate, a forget gate, a cell with a self-recurrent connection, and an output gate. These components together control the information by adding or removing it as necessary^37^. To optimize the LSTM parameters and minimize the loss function, various algorithms like stochastic gradient descent (SGD), Root Mean Square Propagation (RMSProp), and Adaptive Moments (Adam) can be employed. Like RNNs, LSTMs compute a mapping from an input sequence x to an output sequence y by calculating the network unit activations using the following equations iteratively from t = 1 to t = τ with initial values $\:{C}_{o}$ = 0 and $\:{h}_{o}$ = 0. The main difference between an LSTM and a traditional recursive network is that an LSTM cell has a unique memory component C_t_ at each time step. This cell state, along with the weighted input x_t_ and the previous output h_(t−1)_ is used to calculate the new hidden layer output and cell state:

6 $$i_t = \gamma(W_i x_t + U_ih_{t-1} + b_i )$$

7 $$f_t = \gamma(W_fx_t + U_fh_{t-1} + b_f )$$

8 $$o_t = \gamma(W_o x_t + U_oh_{t-1} + b_o )$$

9 $$\widetilde{C_t}= tanh (W_c x_t + U_ch_{t-1} + b_c)$$

10 $$C_t= f_t\odot C_{t-1} + i_t \odot \widetilde{C_t}$$

11 $$h_t= o_t \odot tanh (C_t)$$

where C_(t−1)_ and C_t_ represent the previous and current cell memories, respectively. W_i_: input gates, W_f_: forget gate, and W_o_: output gates. U_i_, U_f_, and U_o_ represent the weight matrices connecting the input, forget, and output gates to the hidden layer respectively. Similarly, b_i_, b_f_, and b_o_ are the bias vectors for the input, forget, and output gates. The (γ) denotes a logistic sigmoid function that is used as a non-linear activation function applied element-wise. The vectors i_t_, f_t_, o_t_, and C_t_ correspond to the input, forget, output gates, and the cell state at any given time stamp t and those are dimensionally equivalent to the cell output vector, h_t_. The operation $\odot$ symbolizes the element-wise multiplication between two vectors. For a more detailed understanding of LSTM networks and their mechanisms, Kratzert et al.^24^, Zhang et al.^39^, Dikshit et al.^40^, and Wang et al.^41^ can provide in-depth discussions and analyses.

### Support vector machine (SVM)

The support vector machine (SVM) was developed in 1992 by Boser et al.^42^, and since then it is commonly employed for classification and regression tasks. Support Vector Machine (SVM) is a supervised learning approach which distinguishes itself by providing a singular, optimal solution for a specific dataset. Conversely, other algorithms may yield several answers in the same context. This characteristic makes SVMs a preferred technique in mitigating overfitting problems by employing a kernel function in nonlinear scenarios to establish decision boundaries^43^. Further, its adaptability has been thoroughly examined, with numerous adjustments producing favorable results^44–46^.

In the context of regression issues, SVM is designated as Support Vector Regression (SVR)^47,48^. The principal objective of SVM is to reduce statistical learning mistakes and improve the model’s stability and robustness^49^. Gunn^50^, Vapnik^51^, and Panahi et al.^52^ provides a brief description of the theory behind support vector regression.

The selection of kernel function affects the performance of SVM models. These functions include; linear, polynomial, radial basis function (RBF), sigmoid, or Gaussian. In this study the Gaussian kernel was selected because of its substantial contribution to model performance. Three critical parameters directly affect the model’s performance with the Gaussian kernel: the scale parameter (γ), the regularization constant (C), and epsilon (ε), as articulated by Belayneh et al.^53^. The parameters were automatically tuned in MATLAB to improve the model’s efficacy. The mathematical formulation of SVM is presented in Eq. (12). The relationship between input and output variables can be distinguished from the equation;

12 $$\:f\left(x\right)=\left(w,\phi\:\left(x\right)\right)+b$$

where f(x) denotes a high dimensional feature space, w represents a weight of the output variable, and b referred as the bias term.

### Extreme gradient boosting (XGBoost)

XGBoost is a powerful machine learning algorithm that has gained significant traction in various fields due to its exceptional predictive performance, particularly in scenarios involving large datasets and complex patterns. Due to the fundamental difference between the boosting and bagging approaches^54^, as a boosting algorithm, where each consequent tree aims to improve the previous ones’ forecast, XGBoost is developed to have lower bias but higher variance when compared to bagging-based models like random forest (RF). Moreover, while still depending on the hyperparameter set, XGBoost is an enhancement of traditional gradient boosting frameworks, incorporating optimizations such as parallel computation, cache awareness, and regularization to mitigate overfitting and enhance model robustness^55^.

Moreover, XGBoost has shown remarkable efficacy in tackling imbalanced (i.e., long-tailed) datasets, a common challenge in atmospheric parameters like wind, precipitation and temperature. For example, Senocak et al.^56^, underlines the XGBoost performance for predicting the daily total precipitation where the dataset includes underrepresented (i.e., extreme) events. Again, in the realm of environmental science, XGBoost was employed to predict PM2.5 concentrations effectively, showcasing its versatility across different contexts^20,57^.

The scalability of XGBoost also contributes to its popularity. By employing a novel sparsity-aware algorithm and weighted quantile sketch for efficient tree learning, it allows practitioners to handle larger datasets without compromising on performance^20^. Its architecture supports parallel computing, making it not only faster but also more efficient, which is crucial in real-time applications like operational weather forecasting^22,58^.

### Categorical boosting algorithm (CatBoost)

CatBoost, an innovative gradient boosting algorithm developed by Yandex^21^, is gaining prominence in machine learning for its unique capability to efficiently handle categorical features. This capability and other design decisions such as ordered boosting leads to significant initial advantages over other traditional gradient boosting techniques such as XGBoost^59^. In a more detailed perspective, CatBoost’s ordered boosting provides a mitigation against the case where the traditional boosting approaches train their models with the entire dataset and this may result in over-optimism in model performance due to possible feature leakage to consequent trees^21^.

Literature indicates that CatBoost exhibits superior performance in various topics of atmospheric research. For instance, a study indicates that CatBoost successfully improve the performances of the best performing numerical weather prediction forecast by up to 72% for atmospheric parameters including precipitation, temperature, and wind over a complex topography spanning across ten different Koppen climate zones and ocean areas. As another instance, a paper focusing on the sub-tropical and sub-humid regions of India compared the performance of CatBoost with various ML methodologies including XGBoost over their predictive performance for predicting weekly pan-evaporation and underlined the performance of the CatBoost^60^.

CatBoost’s architecture contributes to its rapid training and prediction capabilities. This is partly due to its implementation of a symmetric decision tree structure, which allows for faster completion of gradient calculations and enhanced accuracy while mitigating overfitting through the use of mirrored nodes^60–62^.

### Multilayer perceptrons (MLPs)

Multilayer Perceptrons (MLPs) is a widely used neural network model architecture in various fields that require modeling and forecasting time series. The MLP is a feedforward neural network ANN with input, hidden, and output layers^63^. Each layer contains an activation function, which expresses the quantity of output based on the input data mathematically^63,64^. They are widely employed across disciplines, including medical diagnostics, such as predicting heart diseases, where MLPs analyze patient data^65^, and in engineering for applications like fault detection in systems or time series forecasting of energy inputs^66,67^. MLPs use backpropagation-based learning algorithms, adjusting weights across layers to reduce the output errors so that they can effectively model complex and handle the non-linear nature of the relationships^66,68,69^. Furthermore, advancements in optimization techniques, such as Particle Swarm Optimization and Genetic Algorithm integrations, further increase MLP performance in training processes by overcoming local minima and accelerating convergence issues^70^. Their functionality also extends to social sciences, where MLPs assist customer satisfaction predicting research in business environments^71^, indicating their versatility across fields.

### Discrete wavelet transformation

Wavelet Transform is typically presented in two versions in the literature as Continuous Wavelet Transform (CWT) and as Discrete Wavelet Transform (DWT). However, because of the computational complexities associated with the implementation of CWT, DWT is frequently preferred^72–74^. The Discrete Wavelet Transform (DWT) offers an alternative to Fourier transform, decomposing time series data into sub-signals across different frequency components by employing a signal processing, so that the extraction of specific features is enabled^75,76^. It offers a time-frequency analysis of a signal by employing a mathematical function to deconstruct it in the time domain.

The Discrete Wavelet Transform employs a wavelet function, $\:{\psi\:}_{y}$ (t), referred to as the “mother wavelet,” which differentiates among various frequencies. It functions at several scales ($\:{s}_{0}$) and is temporally localized ($\:{\tau\:}_{0}$). The calculation of mother wavelet is presented in Eq. 13:

13 $$\:{\psi\:}_{m,n}\left(t\right)=\frac{1}{{s}_{0}^{m}}\psi\:\left\{\frac{t-{n{\tau\:}_{0}s}_{0}^{m}}{{s}_{0}^{m}}\right\}$$

here m and n indicate controlling parameters of scale and time. The most common selections for the parameters S₀ and τ₀ are 2 and 1, respectively. Based on Mallat’s theory^77^, the Discrete Wavelet Transform (DWT) can decompose a signal into its inverse DWT, resulting in a sequence of approximation and detail signals that are linearly independent. Here, S₀ refers to the step of precision expansion, while τ₀ denotes the location parameter for the DWT applied to a discrete time series x_i_, where each x_i_, occurs at a discrete time i. The inverse DWT, as described by Mallat^77^, is expressed in Eq. (14), which outlines the reconstruction process from these independent signals.

14 $$\:x\left(t\right)=T+\sum\limits_{m=1}^{M}\sum\limits_{t=0}^{{2}^{M-m-1}}{W}_{m,n}{2}^{-\frac{m}{2}}\psi\:\left({2}^{-m}t-n\right)$$

where $\:{W}_{m,n}{2}^{-\frac{m}{2}}{\sum\:}_{t=0}^{N-1}\psi\:\left({2}^{-m}t-n\right)x\left(t\right)$ is the wavelet coefficient for the discrete wavelet at scale $\:s={2}^{m}$ and $\:\tau\:={2}^{m}$. 5 level detailed studies were chosen and employed in wavelet transform in this study since we obtained improved model results. The calculation of the level (L) is based on the Eq. 15.

15 $$\:L=int\left(N\right)$$

where L is the level of the decomposition and N is the number of runs.

In this study, Daubechies45 was preferred among wavelet types such as Haar, Daubechies, and Biorthogonal because it positively affects the model performance.

WT is a data preprocessing method used to estimate the dominant frequency in time series. With this method, the most dominant part of the time series is determined over a certain temporal period. Determining the temporal coverage greatly facilitates forecasting of the time series, because the dominant period in the temporal coverage has an impact over the entire series. Many researchers emphasize that model results have improved, especially with the use of WT in machine learning^78–80^. To combine WT with machine learning methods, analyses are performed after separating the time series into detailed and mother wavelet components at different levels of decomposition. In these analyses, the uniformity of the data type generally has a positive impact on the model results.

### Model performance assessment

The evaluation of model performance was conducted using five recognized statistical metrics: the correlation coefficient (r), root mean square error (RMSE), Nash-Sutcliffe efficiency (NSE), Kling–Gupta efficiency (KGE), and Performance Index (PI). These metrics are defined in Eqs. 16, 17, 18, 19, 20 and 21 respectively.

16 $$\:r=\frac{{\sum\:}_{i=1}^{N}{(x}_{pi}-\underset{\_}{{x}_{p}})\left({x}_{oi}-\underset{\_}{{x}_{o}}\right)}{\sqrt{{\sum\:}_{i=1}^{N}{{(x}_{pi}-\underset{\_}{{x}_{p}})}^{2}}*\sqrt{{\sum\:}_{i=1}^{N}{\left({x}_{oi}-\underset{\_}{{x}_{o}}\right)}^{2}}}$$

17 $$\:RMSE=\sqrt{\frac{1}{N}{\sum\:}_{i=1}^{N}{\left({x}_{oi}-{x}_{pi}\right)}^{2}}$$

18 $$\:NSE=1-\left[\frac{{\sum\:}_{i=1}^{N}{\left({x}_{oi}-{x}_{pi}\right)}^{2}}{{\sum\:}_{i=1}^{N}{\left({x}_{oi}-\underset{\_}{{x}_{o}}\right)}^{2}}\right]$$

19 $$\:KGE=1-\sqrt{{\left(r-1\right)}^{2}+{\left(a-1\right)}^{2}+{\left(\beta\:-1\right)}^{2}}$$

20 $$\:\beta\:=\frac{{x}_{p}}{{x}_{o}},\:\:a=\frac{{\sigma\:}_{{x}_{p}}}{{\sigma\:}_{{x}_{o}}}$$

21 $$\:PI=\frac{RMSE}{\left|{{x}_{p}}\right|}/(1+r)$$

### Model structure

In this study, analyses were conducted using machine learning algorithms. To further investigate model performance and enhance the article’s innovative approach, four different model structures were created by using cross-correlation. The structures of the generated models (time-lagged based on EDI) are shown in Table 2.

**Table 2:** Structure of models (Input lags and forecast target).

Models	Inputs						Output
M01				t-11	t-12	t-1	t
M02			t-13	t-11	t-12	t-1	t
M03		t-2	t-13	t-11	t-12	t-1	t
M04	t-10	t-2	t-13	t-11	t-12	t-1	t

## Results

In this study, monthly rainfall data obtained from three different regions in Norway were first used to calculate the EDI, and then these values were used to make forward predictions using a series of machine learning algorithms. In the analyses, SVM, LSTM, MLP, XGBoost, and CatBoost machine learning algorithms were used. The results obtained from the analyses have been enhanced with wavelet transformation. All the results of the analyses are shown in Table 3. In this table, the best results are shown in bold.

**Table 3:** The results of all models for the regions.

Drammen																	
Before wavelet transformation																	
	SVM						LSTM						MLP				
	*r*	NSE	KGE	PI	RMSE		*r*	NSE	KGE	PI	RMSE		*r*	NSE	KGE	PI	RMSE
M01	0.7656	0.5414	0.3034	1.0997	0.6750	M01	0.7104	0.4893	0.4933	1.1978	0.7123	M01	0.1497	−0.8887	−0.3027	3.4269	1.4162
**M02**	**0.7641**	**0.5424**	**0.3228**	**1.0994**	**0.6743**	M02	0.7691	0.5812	0.5546	1.0488	0.6451	**M02**	**0.5706**	**0.1786**	**0.0741**	**1.6544**	**0.9339**
M03	0.7562	0.5352	0.3507	1.1129	0.6795	**M03**	**0.7708**	**0.5876**	**0.5956**	**1.0397**	**0.6402**	M03	0.3350	−0.7619	0.0745	2.8505	1.3679
M04	0.7575	0.5362	0.3458	1.1109	0.6789	M04	0.7535	0.5562	0.5443	1.0892	0.6641	M04	0.3406	−0.7129	−0.0546	2.7990	1.3487

	XGBoost						Catboost										
	*r*	NSE	KGE	PI	RMSE		*r*	NSE	KGE	PI	RMSE						
M01	0.6200	0.3309	0.1879	1.4476	0.8430	**M01**	**0.6973**	**0.4541**	**0.3227**	**1.2481**	**0.7614**						
M02	0.6178	0.3006	0.0455	1.4820	0.8618	M02	0.6786	0.4144	0.2269	1.3070	0.7886						
**M03**	**0.6411**	**0.3390**	**0.0794**	**1.4203**	**0.8378**	M03	0.6937	0.4325	0.2249	1.2752	0.7763						
M04	0.6409	0.3315	0.0408	1.4286	0.8426	M04	0.6839	0.4092	0.1668	1.3087	0.7921						

After wavelet transformation																	
	SVMW						LSTMW						MLPW				
	*r*	NSE	KGE	PI	RMSE		*r*	NSE	KGE	PI	RMSE		*r*	NSE	KGE	PI	RMSE
M01	0.9095	0.8257	0.8282	0.6267	0.4161	M01	0.9687	0.9378	0.9115	0.3633	0.2487	M01	0.6793	0.4464	−0.1457	3.8154	1.1215
M02	0.9043	0.8138	0.7801	0.6497	0.4301	M02	0.9471	0.8950	0.8750	0.4772	0.3231	M02	0.3973	0.0145	−0.1473	5.2557	1.3574
**M03**	**0.9709**	**0.9416**	**0.9132**	**0.3516**	**0.2409**	M03	0.9735	0.9460	0.8776	0.3377	0.2317	**M03**	**0.8676**	**0.7488**	**0.7937**	**0.7358**	**0.5678**
M04	0.9690	0.9377	0.9248	0.3634	0.2488	**M04**	**0.9765**	**0.9510**	**0.8641**	**0.3211**	**0.2207**	M04	0.8438	0.7040	0.6811	0.7683	0.6626

	XGBoostW						CatboostW										
	*r*	NSE	KGE	PI	RMSE		*r*	NSE	KGE	PI	RMSE						
M01	0.8461	0.7029	0.6083	0.8465	0.5617	M01	0.8746	0.7524	0.6481	0.7610	0.5128						
M02	0.8510	0.6986	0.5212	0.8503	0.5657	M02	0.8831	0.7715	0.7185	0.7278	0.4926						
M03	0.8968	0.7902	0.6963	0.6924	0.4721	**M03**	**0.9236**	**0.8358**	**0.7119**	**0.6039**	**0.4176**						
**M04**	**0.8967**	**0.7906**	**0.7075**	**0.6916**	**0.4715**	M04	0.9182	0.8305	0.7396	0.6154	0.4243						

Hamar																	
Before wavelet transformation																	
	SVM						LSTM						MLP				
	*r*	NSE	KGE	PI	RMSE		*r*	NSE	KGE	PI	RMSE		*r*	NSE	KGE	PI	RMSE
M01	0.7206	0.4734	0.3020	1.1352	0.7233	M01	0.7188	0.5035	0.4905	1.1035	0.7024	M01	0.5452	0.1201	0.2316	1.6340	0.9345
**M02**	**0.7177**	**0.4752**	**0.3267**	**1.1352**	**0.7221**	**M02**	**0.7222**	**0.5047**	**0.4672**	**1.0999**	**0.7015**	M02	0.2918	−1.1474	−0.6264	3.0533	1.4599
M03	0.7153	0.4624	0.2734	1.1506	0.7309	M03	0.7216	0.4971	0.4221	1.1087	0.7069	**M03**	**0.5999**	**0.1922**	**0.3122**	**1.5120**	**0.8953**
M04	0.7186	0.4587	0.2421	1.1523	0.7334	M04	0.7201	0.4968	0.4386	1.1100	0.7071	M04	0.4939	−0.4772	0.3763	2.1898	1.2108

	XGBoost						Catboost										
	*r*	NSE	KGE	PI	RMSE		*r*	NSE	KGE	PI	RMSE						
M01	0.6850	0.4224	0.2937	1.2140	0.7571	**M01**	**0.7072**	**0.4724**	**0.3911**	**1.1452**	**0.7236**						
M02	0.6750	0.4109	0.3353	1.2334	0.7646	M02	0.6995	0.4617	0.3900	1.1620	0.7309						
**M03**	**0.6802**	**0.4259**	**0.3788**	**1.2138**	**0.7548**	M03	0.6901	0.4465	0.3686	1.1849	0.7412						
M04	0.6519	0.3855	0.3346	1.2773	0.7809	M04	0.6815	0.4311	0.3426	1.2073	0.7514						

After wavelet transformation																	
	SVMW						LSTMW						MLPW				
	*r*	NSE	KGE	PI	RMSE		*r*	NSE	KGE	PI	RMSE		*r*	NSE	KGE	PI	RMSE
M01	0.8819	0.7769	0.8099	0.6756	0.4709	M01	0.9529	0.9021	0.7912	0.4313	0.3119	**M01**	**0.6400**	**0.3931**	**−0.0439**	**3.6191**	**1.1404**
M02	0.8834	0.7795	0.8120	0.6711	0.4680	M02	0.9560	0.9121	0.8920	0.4079	0.2955	M02	0.6045	0.3159	−0.7075	4.9156	1.1051
**M03**	**0.9617**	**0.9241**	**0.9102**	**0.3781**	**0.2747**	**M03**	**0.9689**	**0.9369**	**0.8747**	**0.3433**	**0.2503**	M03	0.6670	0.3150	0.5545	0.9484	0.8309
M04	0.9569	0.9134	0.9148	0.4047	0.2933	M04	0.9451	0.8876	0.7840	0.4639	0.3342	M04	0.6554	0.3475	0.5501	1.8062	0.8639

	XGBoostW						CatboostW										
	*r*	NSE	KGE	PI	RMSE		*r*	NSE	KGE	PI	RMSE						
M01	0.8553	0.7265	0.7134	0.7588	0.5210	M01	0.8740	0.7629	0.7971	0.6993	0.4851						
M02	0.8602	0.7295	0.6748	0.7526	0.5182	M02	0.8668	0.7497	0.7758	0.7213	0.4984						
**M03**	**0.8977**	**0.7972**	**0.7538**	**0.6387**	**0.4486**	**M03**	**0.9187**	**0.8375**	**0.7954**	**0.5656**	**0.4016**						
M04	0.8950	0.7898	0.7072	0.6513	0.4568	M04	0.9109	0.8143	0.7064	0.6069	0.4292						

Lillehammer																	
Before wavelet transformation																	
	SVM						LSTM						MLP				
	*r*	NSE	KGE	PI	RMSE		*r*	NSE	KGE	PI	RMSE		*r*	NSE	KGE	PI	RMSE
M01	0.7165	0.4593	0.2599	1.1318	0.7330	M01	0.7184	0.50048	0.47055	1.0866	0.7045	M01	0.5107	−0.1792	0.4323	1.8990	1.0553
M02	0.7195	0.4525	0.2116	1.1368	0.7375	**M02**	**0.7232**	**0.51505**	**0.53577**	**1.0677**	**0.6941**	M02	0.5162	−0.0980	0.0246	1.8259	1.0183
**M03**	**0.7171**	**0.4610**	**0.2673**	**1.1296**	**0.7318**	M03	0.7119	0.49617	0.51004	1.0954	0.7075	**M03**	**0.5827**	**0.1841**	**0.4407**	**1.5078**	**0.8778**
M04	0.7154	0.4441	0.1978	1.1483	0.7432	M04	0.6932	0.46974	0.49723	1.1362	0.7259	M04	0.4417	−0.3649	0.0427	2.1408	1.1354

	XGBoost						Catboost										
	*r*	NSE	KGE	PI	RMSE		*r*	NSE	KGE	PI	RMSE						
M01	0.6498	0.3954	0.5215	1.2451	0.7556	M01	0.7000	0.4649	0.4143	1.1368	0.7109						
M02	0.6344	0.3765	0.5043	1.2764	0.7674	M02	0.6830	0.4427	0.4019	1.1719	0.7255						
M03	0.6579	0.4136	0.4885	1.2203	0.7442	**M03**	**0.7096**	**0.4796**	**0.4125**	**1.1147**	**0.7010**						
**M04**	**0.6633**	**0.4150**	**0.3934**	**1.2148**	**0.7433**	M04	0.6968	0.4608	0.3948	1.1432	0.7136						

After wavelet transformation																	
	SVMW						LSTMW						MLPW				
	*r*	NSE	KGE	PI	RMSE		*r*	NSE	KGE	PI	RMSE		*r*	NSE	KGE	PI	RMSE
M01	0.8626	0.7407	0.7517	0.7223	0.5076	M01	0.9206	0.8364	0.7242	0.5564	0.4032	M01	0.5473	−0.2396	0.3032	1.9011	1.0820
M02	0.8721	0.7588	0.7808	0.6931	0.4896	**M02**	**0.9604**	**0.9209**	**0.8935**	**0.3790**	**0.2804**	M02	0.4754	−0.6028	0.3480	2.2670	1.2303
**M03**	**0.9635**	**0.9258**	**0.8868**	**0.3666**	**0.2716**	M03	0.9505	0.8996	0.8201	0.4291	0.3158	M03	0.8005	0.5018	0.6418	1.0357	0.6860
M04	0.9616	0.9232	0.8967	0.3733	0.2763	M04	0.9550	0.9086	0.8384	0.4086	0.3014	**M04**	**0.8560**	**0.6784**	**0.8293**	**0.8073**	**0.5512**

	XGBoostW						CatboostW										
	*r*	NSE	KGE	PI	RMSE		*r*	NSE	KGE	PI	RMSE						
M01	0.8406	0.6944	0.6258	0.7934	0.5372	M01	0.8396	0.7003	0.7149	0.7862	0.5320						
M02	0.8333	0.6653	0.4932	0.8337	0.5622	M02	0.8486	0.7174	0.7415	0.7598	0.5166						
**M03**	**0.8650**	**0.7302**	**0.6021**	**0.7359**	**0.5048**	M03	0.8807	0.7668	0.7036	0.6784	0.4693						
M04	0.8506	0.7076	0.6000	0.7720	0.5255	**M04**	**0.8907**	**0.7751**	**0.6354**	**0.6626**	**0.4609**						

When examining the results obtained for the Drammen region, in analyses without wavelet transformation, the best performance metrics were achieved in LSTM-M03 (*r* = 0.7708, NSE = 0.5876, KGE = 0.5956, PI = 1.0397, and RMSE = 0.6402). While LSTM yields the best results in this category, SVM-M02 follows closely in performance (*r* = 0.7641, NSE = 0.5424, KGE = 0.3228, PI = 1.0994, and RMSE = 0.6743). Although the performance metrics of these two models are close to each other, the analysis results with LSTM are ahead of SVM. Therefore, in analyses conducted without wavelet transformation, LSTM has demonstrated effective performance compared to other algorithms. In the results obtained with MLP, it was generally found that the NSE (and sometimes KGE) performance metrics were negative. When compared to other methods, MLP is the algorithm with the lowest performance metrics. It is stated by many researchers in the literature that wavelet transformation generally improves the model results^43,81–83^. In this study, wavelet transformation has been applied to all models at 5 detail levels. When these results are examined, just like in the analysis conducted without wavelet transformation, the most successful algorithm has been LSTMW. The performance values of LSTMW-M04 are *r* = 0.9765, NSE = 0.9510, KGE = 0.8641, PI = 0.3211, and RMSE = 0.2207. Although the most successful algorithm before the wavelet transformation is the same (LSTM), the different input data in the model inputs affects the model performance metrics. In other words, before the wavelet transformation, the most successful input structure for LSTM was M03, while after the wavelet transformation, the most successful model input structure was determined to be M04. After the wavelet transformation, significant improvements (almost 100% for some models) were detected for most of the models. As before the wavelet transformation, the second most successful model after the wavelet transformation is SVMW-M03. It has surpassed the other models with the results obtained using LSTM and SVM. It should also be noted that in the analyses conducted with the wavelet transform of MLP, performance metrics such as NSE and r were found to change from negative to positive.

Another region analyzed in this study is Hamar. When examining the results obtained from Hamar, it was found that, as in the Drammen region, the most effective results were achieved with LSTM and SVM in the analysis conducted without wavelet transformation. The performance metrics of LSTM-M02 are *r* = 0.7222, NSE = 0.5047, KGE = 0.4672, PI = 1.0999, and RMSE = 0.7015 while SVM-M02’s performance metrics are *r* = 0.7177, NSE = 0.4752, KGE = 0.3267, PI = 1.1352, and RMSE = 0.7221. These two algorithms are ahead of other machine learning algorithms and models in terms of performance metrics. In this category, although very close results were obtained with the analysis using Catboost-M01, SVM-M02 could not match its performance metrics. The algorithm that performed the worst in this class was MLP. The NSE values of all models except MLP-M03 are negative. Improvements were observed in the results of all models after the wavelet transformation. After the transformation, LSTMW-M03 exhibited the best performance, followed by SVMW-M03 in terms of performance. One of the most notable results after the wavelet transformation is that all the negative values of MLP have become positive. In the wavelet-transformed analysis conducted in this region, the most successful results were obtained with the input structure of M03.

Finally, the region analyzed is Lillehammer. In the analyses conducted before the wavelet transformation, the LSTM algorithm, like in other regions, showed superior performance compared to other models. When the model input structures were examined, it was observed that M02 was successful here. The performance values of LSTM-M02 are *r* = 0.7232, NSE = 0.51505, KGE = 0.5358, PI = 1.0677, RMSE = 0.6941. When comparing the performance metrics of the most successful models in other regions, it has been determined that the values are almost similar to each other. One of the notable results here is that while the second most successful result in other regions is generally achieved with SVM, in Lillehammer, the second most successful performance is achieved with Catboost-M03. In SVM-M03, this model is the second in terms of performance. Again, some parameters of the MLP values obtained within this region have been determined as negative. In the analyses conducted after the wavelet transformation, contrary to other regions, the most successful result was obtained with the SVMW-M03 model. In other regions, it should be noted that the most successful algorithm after the wavelet transform was LSTM. This result has differed from other regions. As in other regions, it has been determined that some NSE values, which were initially identified as negative in the MLP in this region, became positive after the wavelet transformation.

**Fig. 2:**
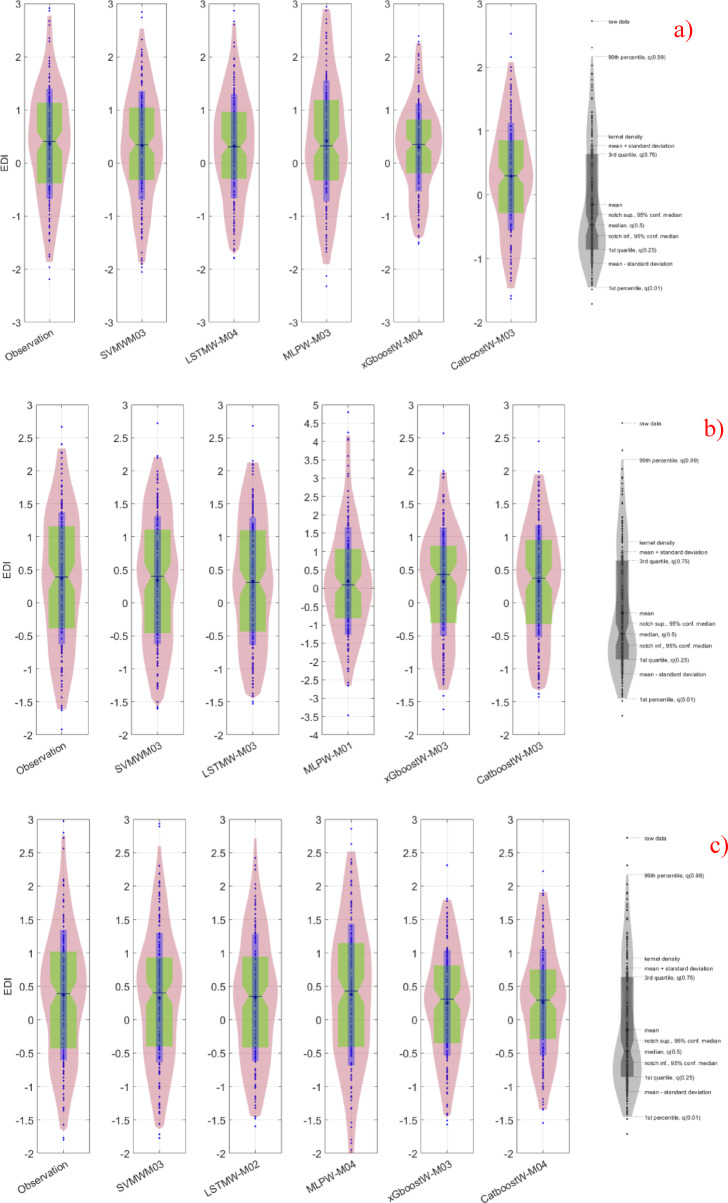
Presentation of the best models (LSTMW-M04, LSTMW-M03, and SVMW-M03, respectively, Drammen, Hamar, and Lillehammer) via Violin diagram in each group where SVMW-M03; analysis of SVM with wavelet transformation for M03, LSTMW-M02; analysis of LSTM with wavelet transformation for M02 etc. and **(a)** Drammen, **(b)** Hamar and **(c)** Lillehammer.

To compare the model results more effectively with each other, the best models in terms of performance metrics were identified and these are shown in the Violin diagram in Fig. 2.

When Fig. 2 is examined for Drammen, it has been determined that the models that show the most similarity to the observed values are LSTMW-M04 and SVMW-M03. Although these two models emerged as the best performers, when examined in detail and considering the average and median values, the most similar model is SVMW-M03. In the statistical results, however, this model is behind LSTMW-M04 in terms of performance. Here, the difference between the statistical results and those obtained from the Violin diagram is noteworthy. In the analysis conducted for Hamar, it was determined that the models most like the observed values were SVMW-M03 and LSTMW-M03. Again, there are very small differences here as well. But it should also be noted that the statistical results of these models are ahead of other models in terms of performance. Therefore, from this perspective, all the results for both regions overlap with each other. A similar situation exists within the Lillehammer region. The performance metrics of the SVM and LSTM algorithms after wavelet transformation are superior to those of the other models. Therefore, within this region, both the statistical results and the visual results are alike one another. In all regions, the morphological differences between the values obtained from the wavelet transformation of the MLP and the observed values are apparent. As a result, in the analyses, the prediction results of MLPW are significantly behind those of the other algorithms. This situation is the same in the statistical results.

**Fig. 3:**
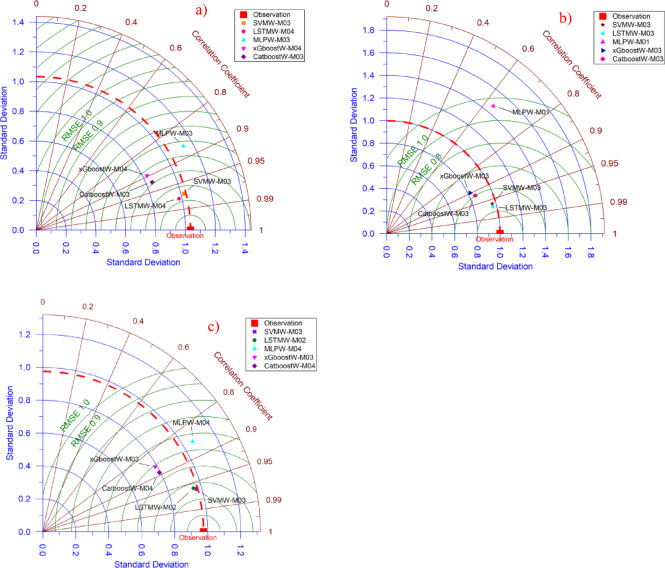
Presentation of the best models (LSTMW-M04, LSTMW-M03, and SVMW-M03, respectively, Drammen, Hamar, and Lillehammer) via Taylor diagram in each group where SVMW-M03; analysis of SVM with wavelet transformation for M03, LSTMW-M02; analysis of LSTM with wavelet transformation for M02 etc. and **(a)** Drammen, **(b)** Hamar and **(c)** Lillehammer.

In Fig. 3, the most successful models in each group are shown in the Taylor diagram. Through this diagram, the model with the best predictive power can be determined by comparing the models. In the analysis conducted for the Drammen region, LSTMW-M04 is the model closest to the observation value. Since the model closest to the observed value in the Taylor diagram exhibited the best performance, it was determined that the most successful model for Drammen is LSTMW-M04. In the Hamar region as well, the most successful model was determined to be LSTMW-M03. In these two regions, the algorithm that follows the most successful models is SVMW. The results obtained from here overlap with the statistical results. In the Lillehammer region, it has been stated in the statistical results that SVMW-M03 shows superiority over LSTM, unlike other regions. The same situation is observed in the Taylor diagram. For this reason, the most successful model for this region has been SVMW. One of the remarkable findings of this study is that this visual comparison method yielded clearer results compared to the Violin diagram.

**Fig. 4:**
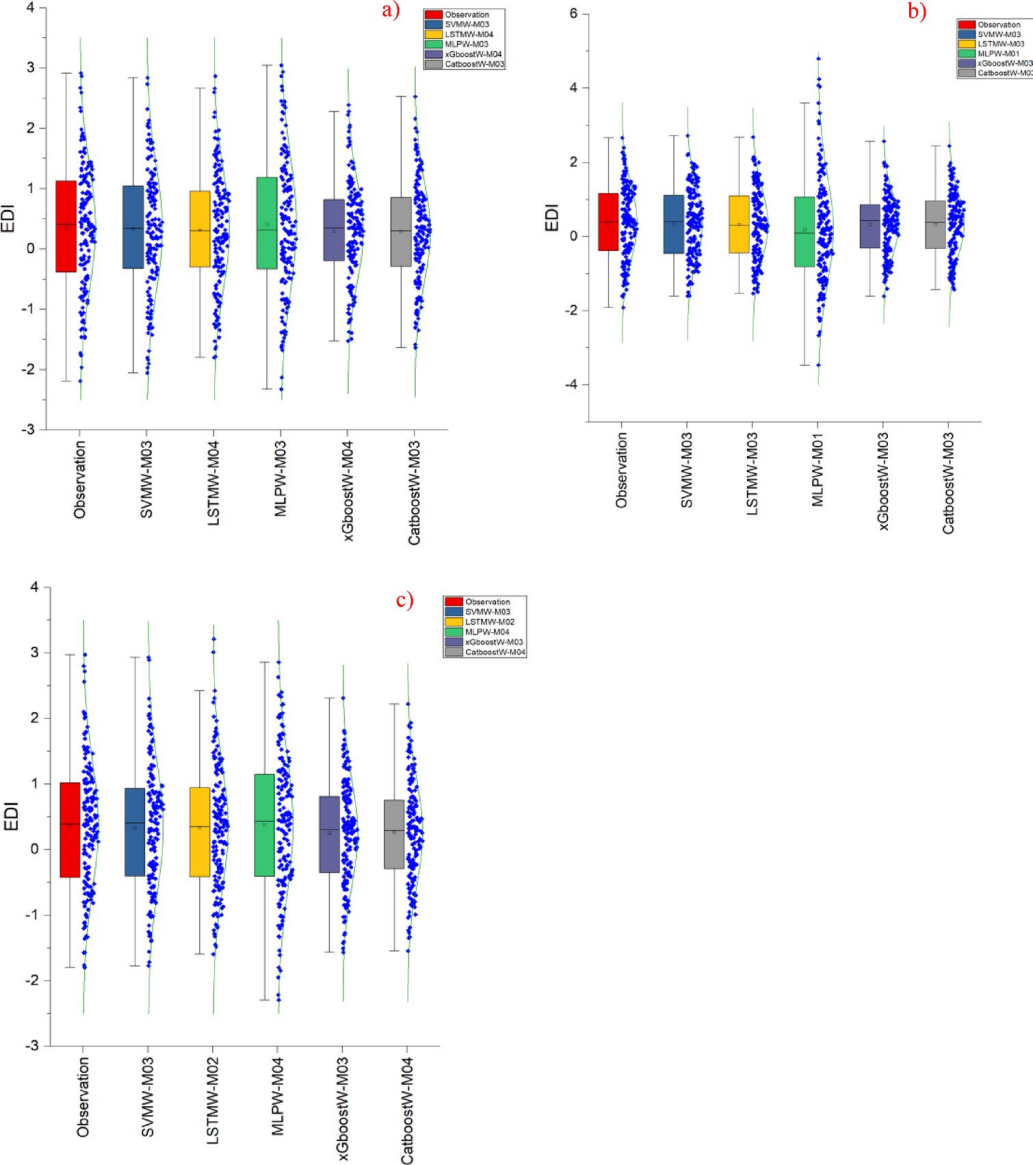
Presentation of the best models (LSTMW-M04, LSTMW-M03, and SVMW-M03, respectively, Drammen, Hamar, and Lillehammer) box-plot (normal overlay) diagram in each group where SVMW-M03; analysis of SVM with wavelet transformation for M03, LSTMW-M02; analysis of LSTM with wavelet transformation for M02 etc. and **(a)** Drammen, **(b)** Hamar and **(c)** Lillehammer.

In Fig. 4, another visual method, the box-plot graph, is compared with the normal distribution through hybridization. Again, the most successful models were selected within their own group, and the best model for each region was determined. As a result of the analysis conducted for Drammen, it was determined that the model consistent with the observed values was SVMW with LSTMW. Since all the predicted and observed values are shown here, they were compared not based on visual similarity but rather on average, outliers, and median values. Considering all these factors, it has been determined that the SVMW-M03 and LSTMW-M04 models have superiority over the other models. In the Hamar region, considering the average, median, and outlier parameters of the observation values, SVMW and LSTMW have shown superiority over the other models. In statistical methods as well, the performance metrics obtained with these two methods are superior to those of the other models. Therefore, the results obtained from this method are similar to those obtained from statistical methods. In the Lillehammer region, however, this situation is somewhat different. Although the superiority of these two models over other methods is obvious, it has been determined that effective results in predicting outliers were not achieved in the analysis conducted with LSTMW. For this reason, the most successful algorithm and model in this region is SVMW-M03. All the results obtained with this analysis method overlap with the statistical results.

**Fig. 5:**
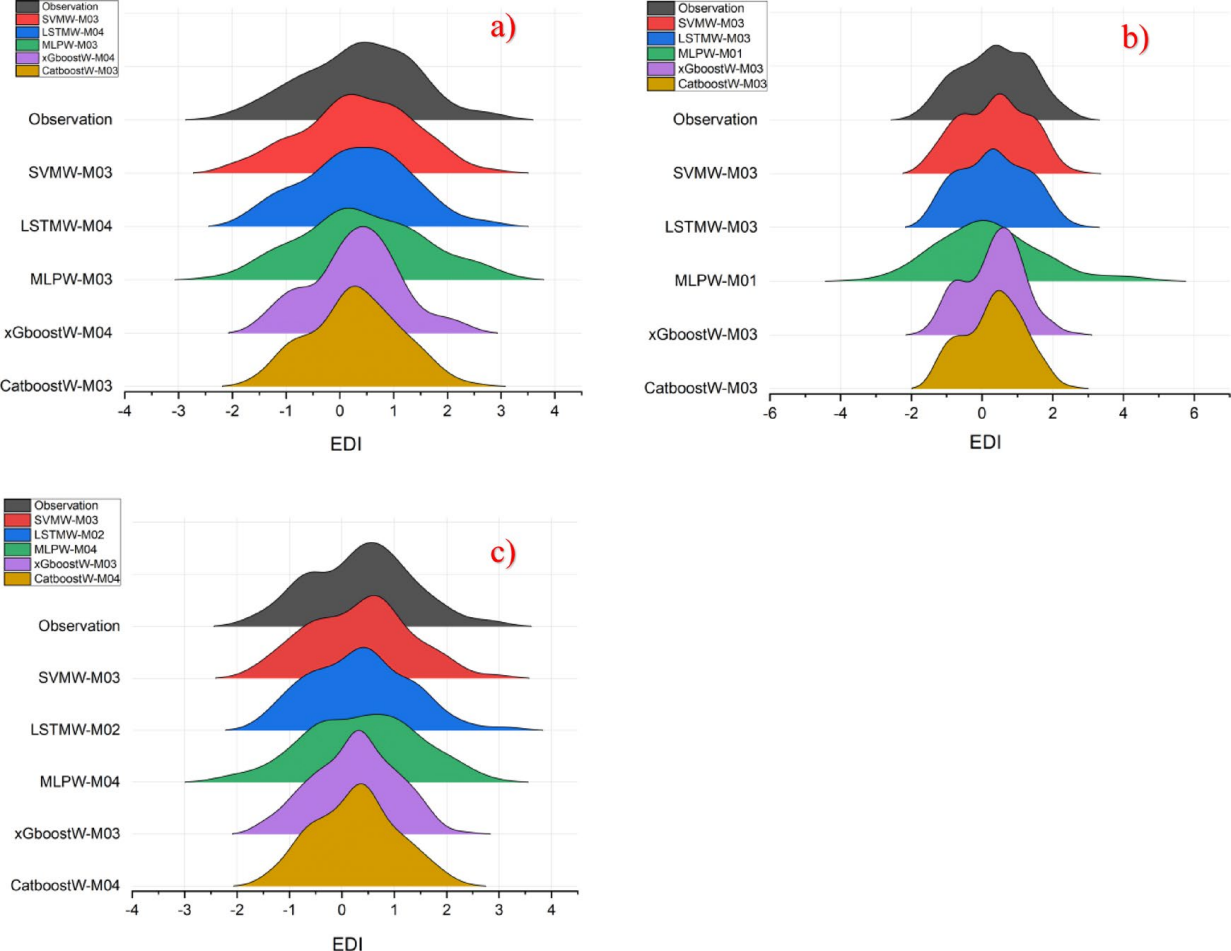
Presentation of the best models (LSTMW-M04, LSTMW-M03, and SVMW-M03, respectively, Drammen, Hamar, and Lillehammer) ridge diagram in each group where SVMW-M03; analysis of SVM with wavelet transformation for M03, LSTMW-M02; analysis of LSTM with wavelet transformation for M02 etc. and **(a)** Drammen, **(b)** Hamar and **(c)** Lillehammer.

Figure 5 shows the ridge diagram of the most successful models. According to the results obtained from this diagram, for the Drammen region, the models that are most successful in predicting peak points and are visually most similar to the observed values are SVMW-M03 and LSTMW-M02. In the Hamar region as well, the most successful models are the LSTMW-M03 and SVMW-M03 models, just like in Drammen. In these visuals, the shape differences and peak points are the most important factors in identifying the most successful model. The most successful algorithms for the Lillehammer region are SVMW-M03 and LSTMW-M02. All the results obtained from here overlap with the statistical results.

**Fig. 6:**
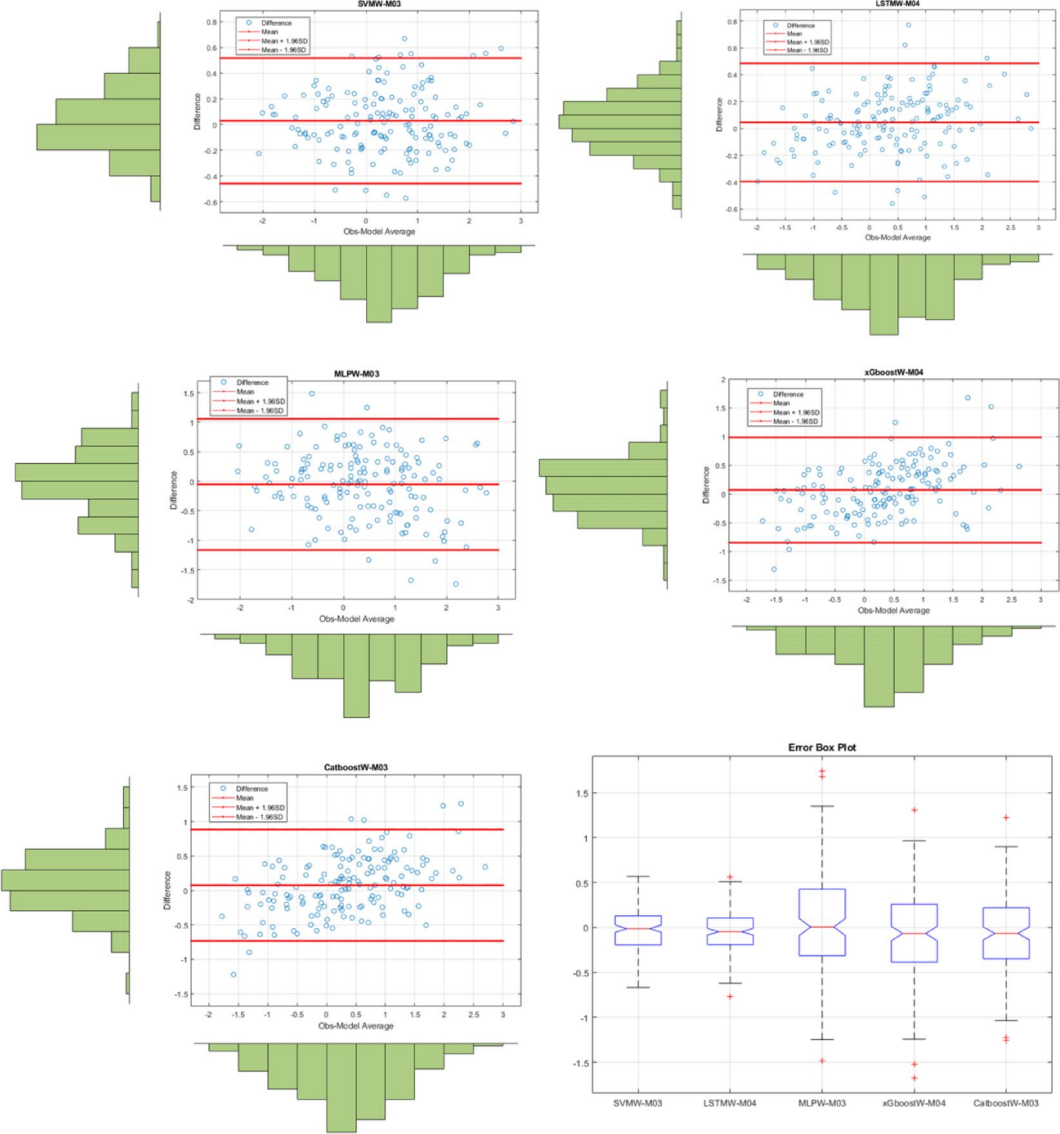
The results of all models on the Bland-Altman diagram and the Error Box diagram for Drammen where SVMW-M03; analysis of SVM with wavelet transformation for M03, LSTMW-M02; analysis of LSTM with wavelet transformation for M02 etc.

**Fig. 7:**
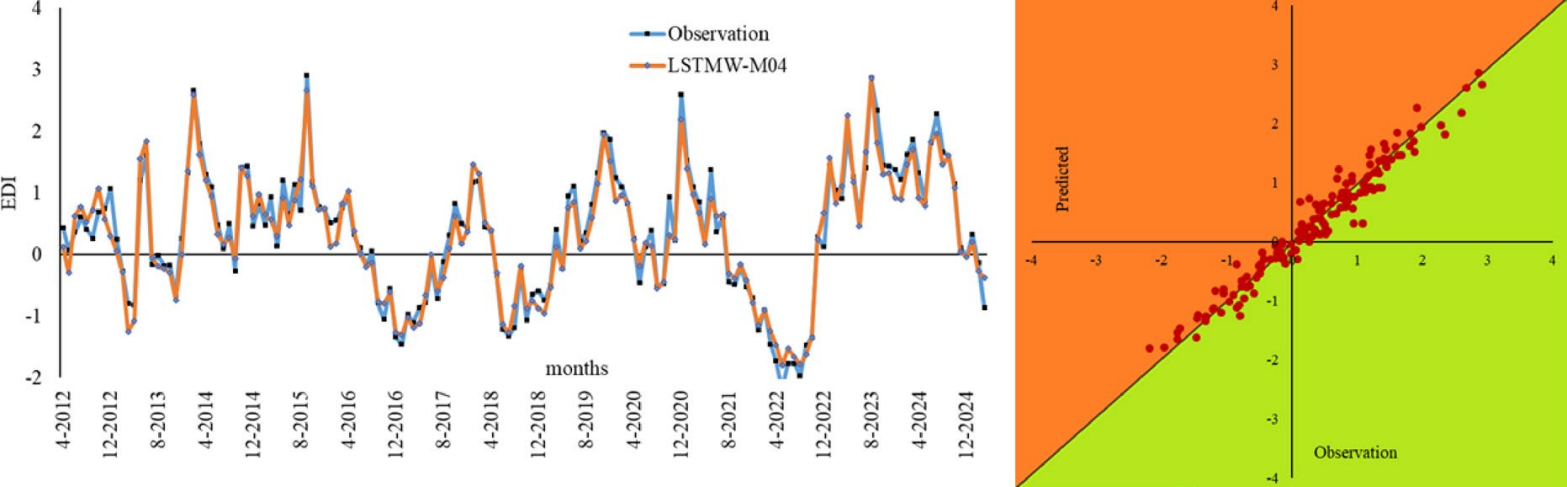
Time series of the best models in Drammen and scatter diagram.

 Figure 6 shows the Bland-Altman and Error Box plots for Drammen. When these graphs, created based on observation values, are examined, the small distances between the difference values within the limits of the Bland-Altman graphs, due to their basis on error values, indicate the best model to us. Therefore, in the Drammen region, the boundaries corresponding to the ± 1.96 significance level (limits of agreement) in the LSTMW-M04 model provide the best performance compared to the other models. This makes this model the best-performing model. In Fig. 7, the time series representation and scatter diagram of this model are provided. When this figure is examined, it has been determined that the observed values generally overlap with the predicted values.

**Fig. 8:**
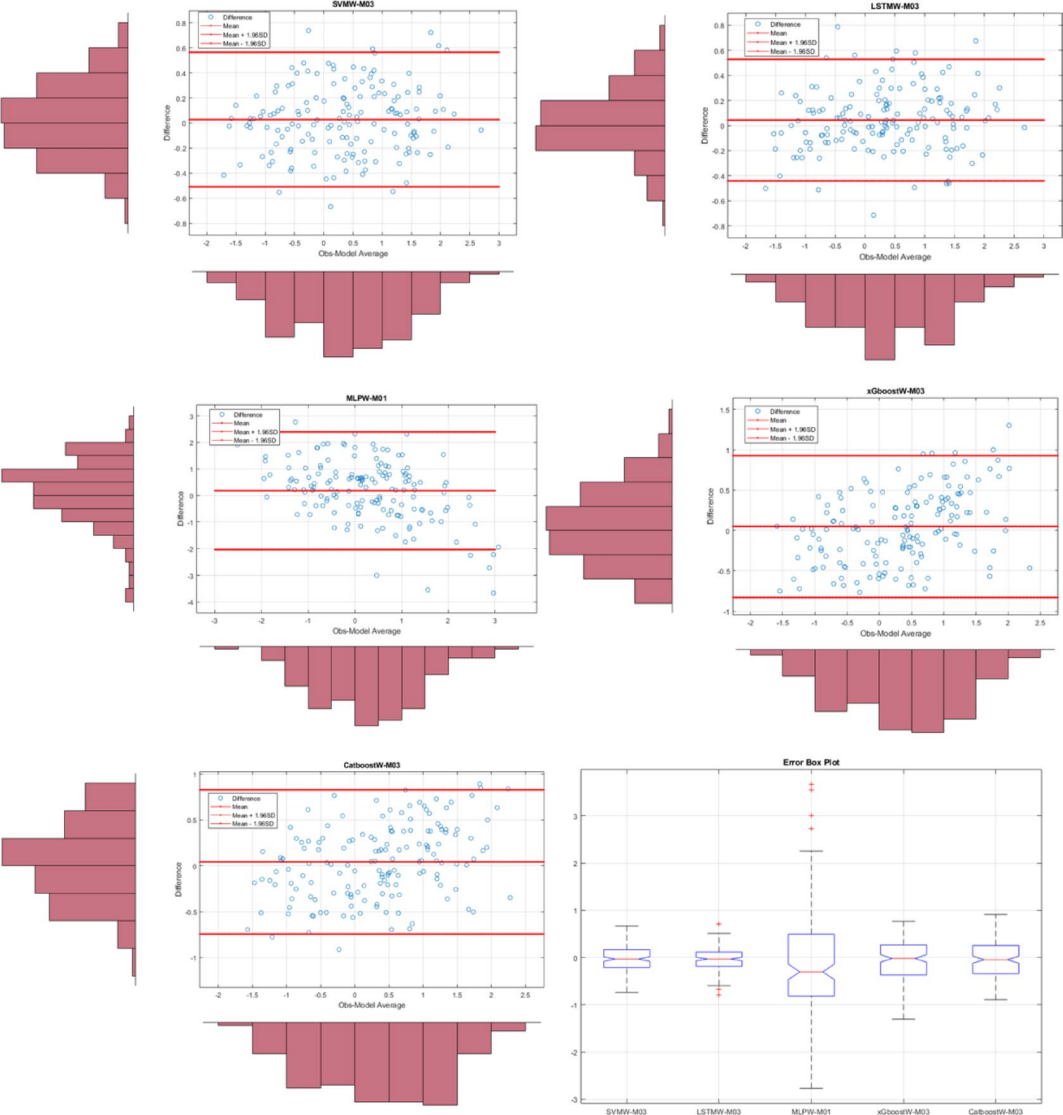
The results of all models on the Bland-Altman diagram and the Error Box diagram for Hamar where SVMW-M03; analysis of SVM with wavelet transformation for M03, LSTMW-M02; analysis of LSTM with wavelet transformation for M02 etc.

**Fig. 9:**
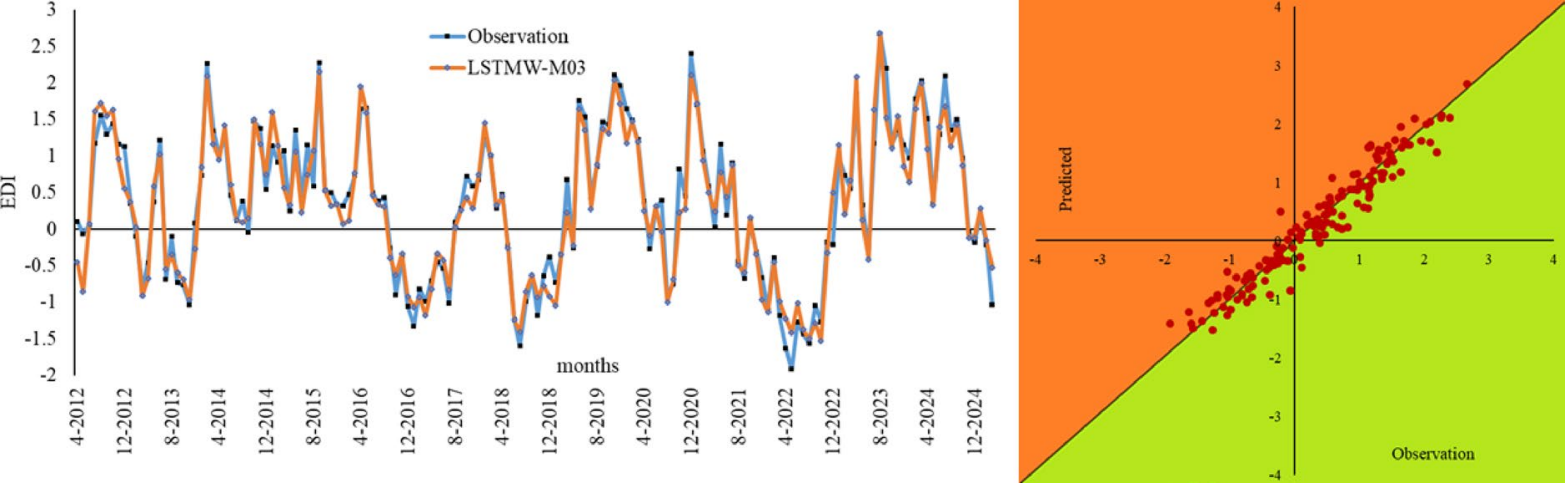
Time series of the best models in Hamar and scatter diagram.

In Fig. 8, Bland-Altman and Error Box plots for Hamar are shown. Bland-Altman graphs were created based on the observed values for each model. To enhance the model comparison, an Error box plot has been drawn. The model with the shortest distance between the difference values created based on the ± 1.96 significance levels (limits of agreement) offers the best model performance. Accordingly, the best result for the Hamar region was obtained with the LSTMW-M03 model. Additionally, when examining the Error box plot, it was concluded that the error rate was lowest for all values except for the outliers. The same result was obtained in both graphs. The time series and scatter diagram for this model are shown in Fig. 9. When this figure is examined, it has been identified that the observed values generally overlap with the predicted values.

**Fig. 10:**
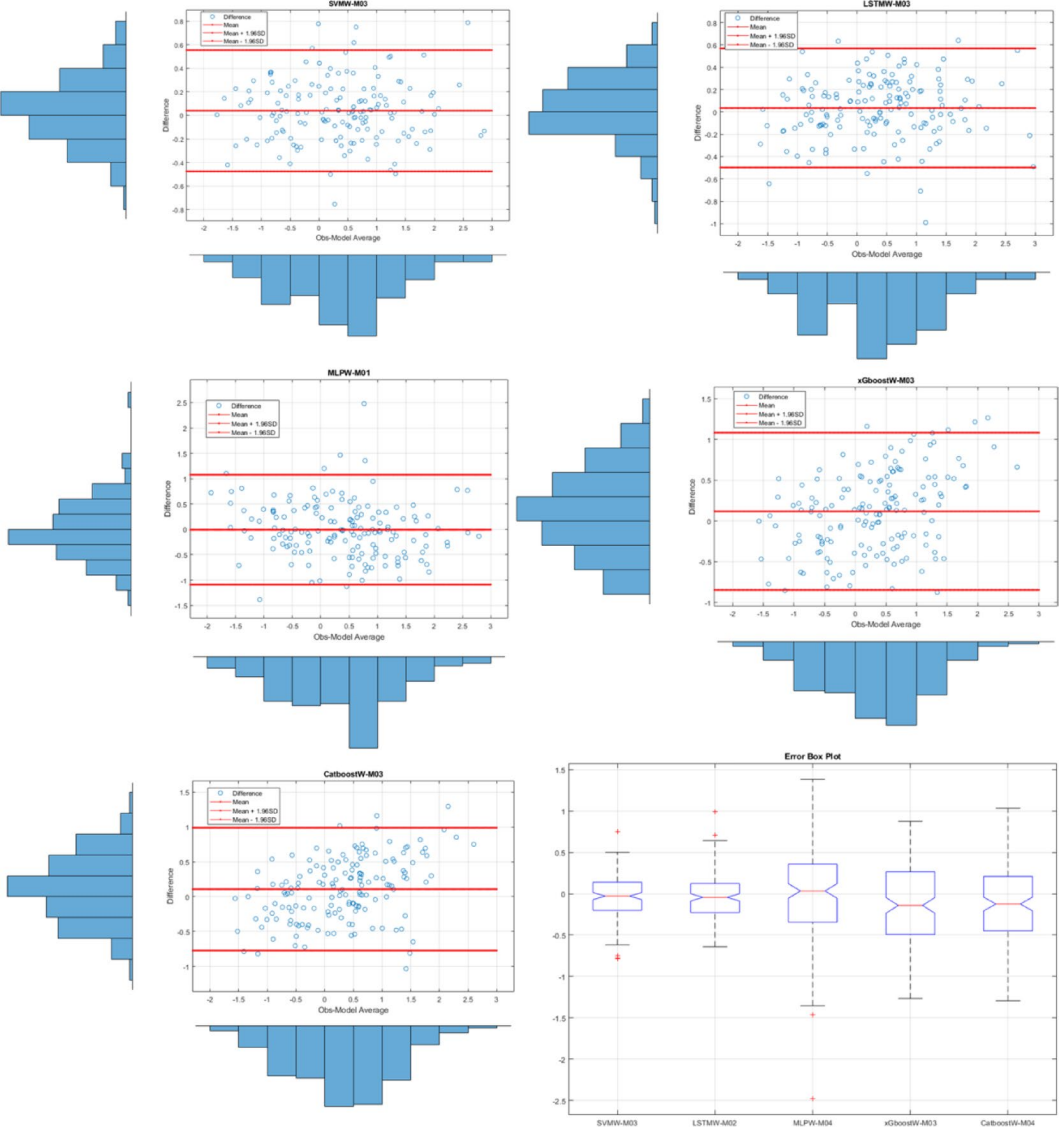
The results of all models on the Bland-Altman diagram and the Error Box diagram for Lillehammer where SVMW-M03; analysis of SVM with wavelet transformation for M03, LSTMW-M02; analysis of SVM with wavelet transformation for M02 etc.

**Fig. 11:**
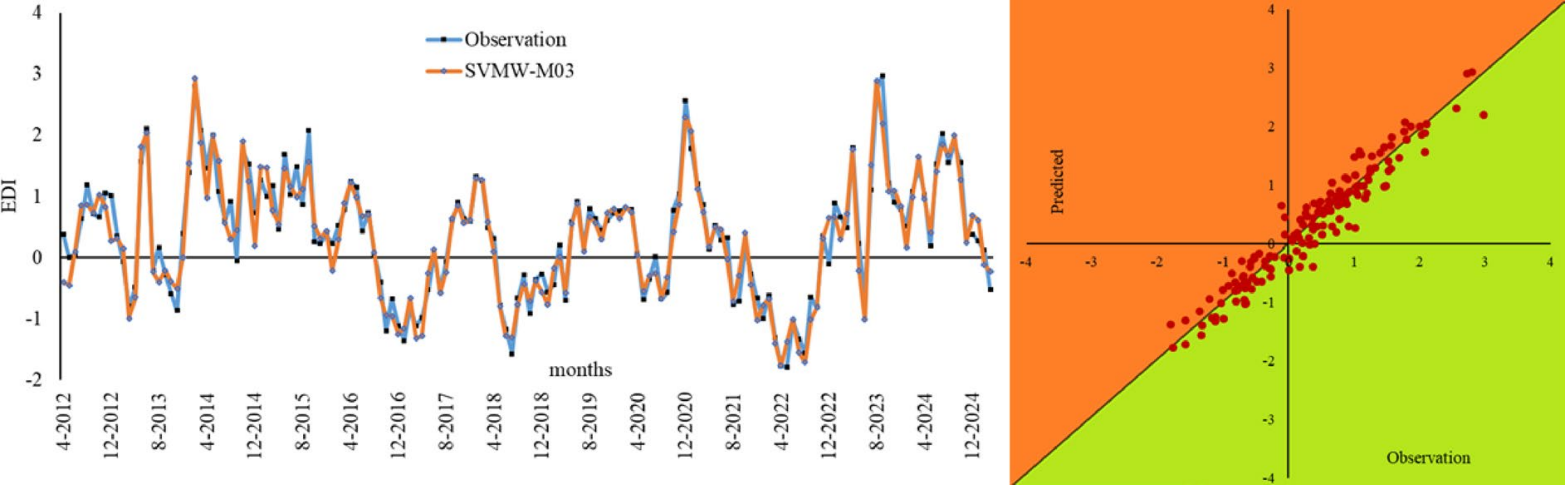
Time series of the best models in Lillehammer and scatter diagram.

Figure 10 shows the Bland-Altman and Error Box plots for Lillehammer. In the analyses conducted for this region, the best performance was exhibited by SVMW-M03. Although the LSTMW-M03 model showed similar results, it was determined that SVM exhibited superior performance with small differences, and this situation remained unchanged in the Error Box graph. The results obtained from these graphs, which are based on observation values, overlap with the statistical results. The time series and scatter diagram belonging to SVMW-M03, which has the best performance metrics in Lillehammer, are shown in Fig. 11. When this graph is examined, the observed values generally overlap with the predicted values.

For each region, we examined the mean difference between the top-performing model’s predictions and the test-set data. We used one-way ANOVA and also applied the Kruskal–Wallis test. In all cases, the p-values were greater than 0.05; therefore, H₀ (no difference) was not rejected (Table 4).

**Table 4:** Statistical significance (ANOVA / Kruskal–Wallis) for the top-performing models.

	F	Observed significance	H0
Drammen			
ANOVA	0.15152	0.6973	Not Rejected
Kruskal-Wallis	-	0.6183	Not Rejected
Hamar			
ANOVA	0.1504	0.6984	Not Rejected
Kruskal-Wallis	-	0.6289	Not Rejected
Lillehammer			
ANOVA	0.1277	0.7210	Not Rejected
Kruskal-Wallis	-	0.7423	Not Rejected

## Discussion

This study calculated monthly rainfall and EDI values obtained from Drammen, Hamar, and Lillehammer in the Norway region, and subsequently created different input structures with these values to be used in machine learning methods. These input structures were analyzed using SVM, LSTM, MLP, XGBoost, and CatBoost machine learning algorithms, and the obtained results were enhanced with wavelet transformation. 70% of the dataset was used for training, and 30% for testing. In this study, the performance metrics used to compare the models were derived from the test data.

The findings obtained from the study are consistent with the results of similar studies in the literature. Some of these include: Tuğrul et al.^81^ obtained precipitation data from a meteorological station near the Apa Dam located in the Konya Closed Basin, first calculating the SPI values for the region, and then attempting to predict these values using machine learning. They used machine learning methods such as SVM and LSTM. To strengthen the model results, they utilized the wavelet transform technique. In their findings, they emphasized that both methods showed superior performance and that the model results improved after the wavelet method in their study. In this study, improvements in model results were also detected using the wavelet method. Danandeh Mehr et al.^84^ conducted analyses using the SPEI data calculated from two different stations in Ankara, employing LSTM, Genetic Programming (GP), Convolutional Neural Network (CNN), and ANN methods. They have strengthened their analyses by hybridizing some of these methods. In their findings, they stated that the CNN-LSTM method outperformed other methods and that LSTM also performed well on its own. In this study, wavelet transformation, referred to as a data preprocessing method or hybrid method, was used. Additionally, good performance metrics were achieved in the results obtained with both LSTM and LSTMW. As a result, the findings obtained from the two studies overlap with each other. In another study where LSTM was used for forward-looking drought prediction, it is Taylan’s^85^ work. In Taylan’s study, some stations located in the Sakarya Basin first calculated SPI values using the precipitation data they obtained and then conducted forward-looking forecasting studies with the help of LSTM. In the study, different input structures were determined using autocorrelation, and analyses were conducted based on the most suitable model input structure. In this study, unlike Taylan^85^, the most suitable input structure was determined using cross-correlation, and model diversity was created. Just like in this study, it is mentioned in the findings of Taylan^85^ that good performance metrics were achieved with LSTM. Coşkun and Citakoglu^86^ conducted a study comparing the performance of ELM and LSTM. In their study, they first calculated the SPI using data obtained from the Sakarya station and then conducted analyses using machine learning methods. They have stated in their findings that LSTM yields better results compared to ELM. In this study, it has been determined that LSTM is more effective compared to other methods.

One of the most important points that distinguishes this study from others in the literature is the use of EDI as input data. Making drought predictions using machine learning has become one of the quite popular topics recently. In these studies, researchers generally prefer SPI or SPEI as the drought index^43,87–91^. In this study, EDI was preferred, unlike the studies in the literature. Because EDI can provide better drought resolutions compared to SPI^17^. With LSTM, not only droughts but also the prediction of hydrological and meteorological parameter is being made in the literature. In the findings obtained from these studies, it is generally stated that the model performances are at a satisfactory level^92,93^.

In data-driven machine learning methods, data preprocessing processes generally improve model performance. These can be optimization techniques as well as WT. When these studies in the literature are examined, the analyses generally show positive impacts on model performance metrics^43,88,94^. However, in some studies, especially in tree-based models, performance metrics may be negatively affected^94^. In WT methods, parameters that can affect model performance metrics vary depending on the wavelet level and wavelet type. The daubechies45 wavelet type commonly yields the most effective results. Many researchers also prefer wavelet as a method^94–96^.

Finally, we provide a table of hyper-parameters for all the machine learning algorithms we used in this study in Table 5. The values in this table have been adjusted to best predict the model outcome. Analysis results can be evaluated by trying different parameter values.

**Table 5:** Hyperparameters for all algorithms.

	LSTM	MLP	Catboost	XGboost	SVM
Model nodes	2	-	-	-	-
Epoch	25–35-45	-	-	300	-
Interpolate method	linear	-	-	-	-
Optimizer	ADAM	-	-	-	-
Learning rate	0.05	-	0.05	0.05	-
Iterations	-	-	300	-	-
Activation	-	Relu	-	-	-
Solver	-	Adam	-	-	-
Lambda	-	0.0001	-	-	-
Alpha	-	Constant	-	-	-
Gamma	-	-	-	0.01	-
Hidden layer	-	1 to 4	-	-	-
Depth	-	-	4	4	-
Random state	-	-	-	42	-
Decision Func.	-	-	-	-	one-and-one
Kernel Func.	-	-	-	-	rbf
Kernel coefficient	-	-	-	-	0.001–1.001
Random seed	-	-	42	38	-
Shuffle	No	No	No	No	No

## Conclusion

In this study, the results obtained from different machine learning methods were presented using four different model input structures. The most significant results obtained are expressed below:

- WT improved performance metrics across all algorithms and nearly all input configurations, demonstrating its positive effect.
- For Drammen, the most successful result both before and after the wavelet was detected using the LSTM algorithm. Additionally, it has been determined that the most successful model input structure for this region is M04. In a modeling study to be conducted here, it is recommended to use the LSTMW-M04 model input structure.
- In the analyses conducted in the Drammen region with MLP, the r and KGE performance metrics were determined to be negative without wavelet transformation, but after wavelet transformation, these performance metrics became positive. MLP is the algorithm that performs the worst in this region.
- In the analysis conducted without wavelet transformation in the Hamar region, the best performance metrics were obtained in the LSTM’s M02 model. As in Drammen, the best performance in this region was also detected in LSTM, while the worst performance was detected in MLP. Additionally, SVM follows LSTM in terms of performance.
- In Hamar, the CatBoost algorithm demonstrated performance close to SVM, creating one of the striking results.
- In Hamar, the model inputs for the most successful models were obtained with M03. In any analysis conducted in this region, this input structure should be used.
- The CatBoost and XGBoost algorithms did not achieve the performance metrics of LSTM and SVM either before or after the wavelet transformation.
- In Lillehammer, unlike other regions, the most successful result before the wavelet transformation was achieved with LSTM-M02, while the second most successful result was obtained with Catboost-M03. Additionally, the most successful result after the wavelet transformation was obtained with SVMW-M03. In other regions, the most successful results before wavelet transformation are generally obtained with LSTM, whereas here, obtaining them with SVM is one of the most striking results of the study. This also shows that the algorithm used can yield different results in different regions.
- In the study, the most effective performance in all regions was achieved in the Drammen region with LSTMW-M04. This algorithm and model input structure should be used for future forecasting models in this region.
- Overall, LSTM wavelet transformation has provided superiority over other methods.
- The results obtained from the wavelet transform of MLP exhibited the weakest performance in all regions.
- Although MLP is a powerful one, MLP results are generally negative and low due to the high nonlinearity in the datasets. Our claim here is that WT improves model performance. With WT, seasonality, trends, and extreme points in the dataset are more clearly differentiated and accurately estimated.

With this study, the most effective models and algorithms for detecting future droughts in the working areas of Drammen, Hamar, and Lillehammer in Norway have been identified. In a future modeling study to be conducted in the region, the results and findings obtained from this study will serve as a guiding reference for the upcoming work. Additionally, this study is limited regionally in terms of the algorithms used, the data used, and the data preprocessing method.

This study will contribute to future regional agricultural policies and policies for water-based energy production facilities, as these policies are directly affected by drought. Therefore, the most appropriate drought model for each region has been determined here to help predict droughts and assist decision-makers.

The data obtained from the study area, the machine learning techniques used, and the data preprocessing methods applied can be mentioned among the limitations for this study. Future studies could improve model performance and obtain more effective results by using different data preprocessing methods and adding different meteorological and hydrological parameters to the input data such as temperature, streamflow.

## Data Availability

The original contributions presented in the study are included in the article. The raw data supporting the conclusions of this article will be made available by the corresponding authors upon reasonable request.
